# Improvement of Drug Release and Compatibility between Hydrophilic Drugs and Hydrophobic Nanofibrous Composites

**DOI:** 10.3390/ma14185344

**Published:** 2021-09-16

**Authors:** Hazim J. Haroosh, Yu Dong, Shaimaa Jasim, Seeram Ramakrishna

**Affiliations:** 1 School of Civil and Mechanical Engineering, Curtin University, Perth, WA 6845, Australia; hazim.haroosh@curtin.edu.au; 2 Department Biomedical Science, Murdoch University, Perth, WA 6150, Australia; 32862216@student.murdoch.edu.au; 3 Department of Mechanical Engineering, National University of Singapore, Singapore 117575, Singapore; seeram@nus.edu.sg

**Keywords:** electrospun nanofibres, nanocomposites, polylactic acid (PLA), poly(*ε*-caprolactone) (PCL), halloysite nanotubes (HNTs), drug release, release kinetic modelling

## Abstract

Electrospinning is a flexible polymer processing method to produce nanofibres, which can be applied in the biomedical field. The current study aims to develop new electrospun hybrid nanocomposite systems to benefit the sustained release of hydrophilic drugs with hydrophobic polymers. In particular, electrospun hybrid materials consisting of polylactic acid (PLA):poly(*ε*-caprolactone) (PCL) blends, as well as PLA:PCL/halloysite nanotubes-3-aminopropyltriethoxysilane (HNT-ASP) nanocomposites were developed in order to achieve sustained release of hydrophilic drug tetracycline hydrochloride (TCH) using hydrophobic PLA:PCL nanocomposite membranes as a drug carrier. The impact of interaction between two commonly used drugs, namely TCH and indomethacin (IMC) and PLA:PCL blends on the drug release was examined. The drug release kinetics by fitting the experimental release data with five mathematical models for drug delivery were clearly demonstrated. The average nanofiber diameters were found to be significantly reduced when increasing the TCH concentration due to increasing solution electrical conductivity in contrast to the presence of IMC. The addition of both TCH and IMC drugs to PLA:PCL blends reduced the crystallinity level, glass transition temperature (*T_g_*) and melting temperature (*T_m_*) of PCL within the blends. The decrease in drug release and the impairment elimination for the interaction between polymer blends and drugs was accomplished by mobilising TCH into HNT-ASP for their embedding effect into PLA:PCL nanofibres. The typical characteristic was clearly identified with excellent agreement between our experimental data obtained and Ritger–Peppas model and Zeng model in drug release kinetics. The biodegradation behaviour of nanofibre membranes indicated the effective incorporation of TCH onto HNT-ASP.

## 1. Introduction

Electrospinning, as a combination of two processing methods, namely electrospraying and spinning [1,2], is an efficient, convenient and versatile method for creating reliable ultrafine fibres from polymer solutions. It has been a hot subject with significant growing interest to scientific communities during recent years. It offers a promising technique to manufacture continuous fibres with the diameters ranging from nanometres to microns [3,4]. It is also deemed as an alternative approach to fabricate hybrid fibre membranes based on polymer nanocomposites when compared with commonly used manufacturing processes of twin-screw extrusion, high shear mixing and injection moulding. Polymer nanocomposites when incorporated with nanoparticles become very attractive advanced materials due to their well-tailored properties [5] and unique structures, which might not be possibly accomplished in conventional composites [6,7]. Halloysite nanotubes (HNTs) have also gained widespread attention as tubular clay-based nanoparticles with cost-effectiveness mainly used for the reinforcement purpose [8]. In general, poly(*ε*-caprolactone) (PCL) is a hydrophobic and semi-crystalline polymer [9]. It degrades in the body due to the existence of an aliphatic ester bond for hydrolysis [10], and the resulting materials can be metabolised via tricarboxylic acid cycle or eliminated directly by the renal secretion [11]. On the other hand, polylactic acid (PLA), as one of the most promising biodegradable polymers, has remarkable material merits including easy processability, ability to be dissolved in common solvents, good sustainability and reasonable biocompatibility [12,13]. Since PLA has a low degree of crystallinity, when compared with PCL, which can degrade to form nontoxic monomers, it is well utilised in biomedical applications such as medical devices, drug delivery, tissue scaffolding, etc. [14]. As a typical example, it submits the secession to monomeric units of lactic acid in the body, which naturally takes place in the carbohydrate metabolism [15,16]. Whereas, PCL is a slowly degraded biopolymer as opposed to many other counterparts such as PLA owing to its semi-crystalline nature [17,18] and a high degree of crystallinity. Moreover, PCL is not harmful to local tissues because it does not form an acidic environment like poly(lactide-co-glycolide) (PLGA) or PLA. In the past decades, the use of polymers, as a carrier and release controller for drug delivery systems, has been the major research focus due to the improvement of healing effectiveness and the reduction in toxic side-effect [19]. In addition, the interaction between drugs and their carriers is paramount to control a sustained drug release [20,21]. Hydrophobic drugs including clindamycin, ciprofloxacin, and cephalexin can be simply incorporated with, and thus interacted with hydrophobic polymers. However, hydrophilic drugs cannot be embedded into hydrophobic polymers due to their weak interactions while the drug release rate may not be well controlled [22]. This study aims to develop new electrospun hybrid nanocomposite systems to benefit the sustained release of hydrophilic drugs when encountered with hydrophobic polymers in order to ultimately overcome the weak interaction issue identified between the drugs and their carriers. 

## 2. Materials and Methods

### 2.1. Materials

**Figure 1:**
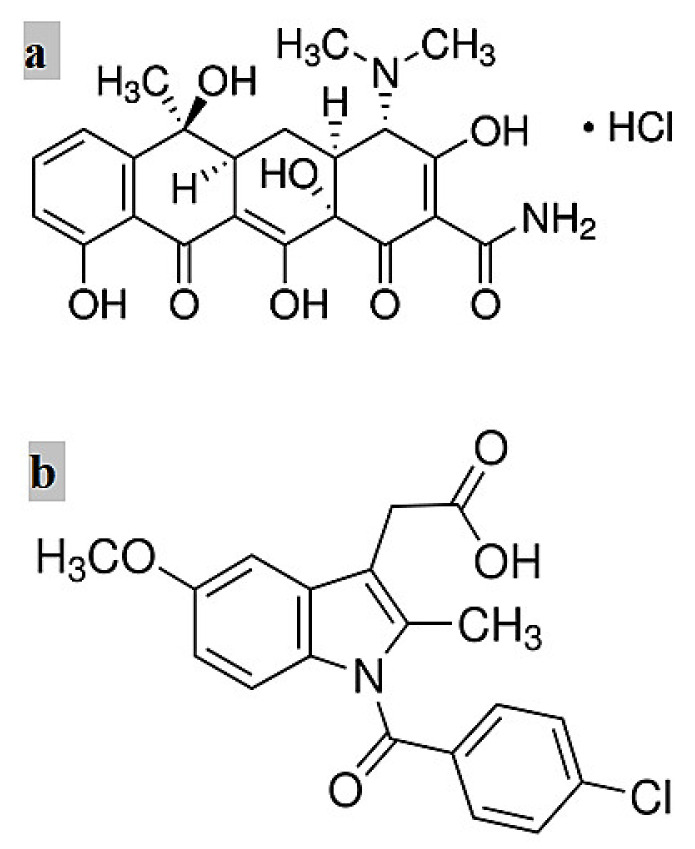
Chemical structure of two model drugs: (**a**) TCH [23] and (**b**) IMC [24].

PLA 3051D (molecular weight (*MW*) = 93,500 g/mol) was supplied by Nature Works, Blair, Nebraska, USA. PCL (*MW* = 80,000 g/mol), tetracycline hydrochloride (TCH) (C_22_H_24_N_2_O_8_·HCl, *MW*= 480.9 g/mol) shown in Figure 1a, Indomethacin (IMC) (C_19_H_16_ClNO_4_, MW = 357.79 g/mol) illustrated in Figure 1b, phosphate buffer solution (PBS), chloroform and methanol were all purchased from Sigma-Aldrich Ltd., Castle Hill, NSW, Australia, and these materials were used without any purification. Halloysite nanotubes (HNT) were obtained from Imerys Ceramics, Kaeo, Northland, New Zealand.

### 2.2. Electrospinning

TCH and IMC drugs with a fixed concentration of 5 wt% were initially mixed with blend solutions (i.e., PLA:PCL at a mix ratio of 1:1), in which TCH was mixed with 1wt%/v HNT-ASP and subsequently added to PLA:PCL solution. The solvent used in all cases was the mixture of chloroform and methanol (volume ratio: 2:1). In the following electrospinning process, different types of solutions were transferred to a 10 mL syringe pump (A Fusion 100 syringe pump, Chemyx Inc., Stafford, TX, USA) with a needle specification of 20 G (inner diameter: 0.584 mm). The flow rate of the solution was set to 2 mL/h, the applied voltage was in the range of 25–28 kV, and the needle-to-collector distance was fixed at 13 cm. The electrospinning process was carried out at 24 °C in the closed system and in ventilated fume cupboards with optimal conditions of humidity and temperature. The polymer solution was electrospun immediately after being prepared to reduce the effect of environmental conditions. The resulting fibres were collected on a ground collector covered by flat aluminium foil. A sharp blade was used to cut fibre mats and they were removed with fine forceps. The thicknesses of fibre mats produced were measured using a digital electronic micrometer, recorded in the range from 330 to 450 µm. 

### 2.3. In Vitro Drug Release Study

The drug-loaded fibre mat samples (sample size: 2 cm × 2 cm) were incubated in 20 mL PBS (pH = 7.4) using a rotary shaker at 37 °C. After the required incubation time for drug release, the samples were transferred to 20 mL fresh buffer solution and 36 separate incubations were conducted to obtain TCH and IMC release data for the three nanofibre mats (i.e., PLA:PCL/IMC, PLA:PCL/TCH and PLA:PCL/HNT-ASP/TCH) and subsequently, the released drug amount in the buffer solution was determined accordingly.

The percentage of the released drug was calculated from the primary weight of the drug (i.e., TCH and IMC) contained in the electrospun mats. The cumulative amount of drug released from nanofibre mats was calculated according to the following Equation:

(1) $$\mathrm{Cumulative}\;\mathrm{amount}\;\mathrm{of}\;\mathrm{drug}\;\mathrm{released}\;\left(\%\right)=\left(\frac{M_{t}}{M_{\infty }}\right)\times 100\%$$

where *M_t_* is the number of drugs released up to time *t* and *M_∞_* is the initial amount of drug within electrospun fibres.

### 2.4. Mathematical Models for Drug Release Kinetics

The kinetics of TCH and IMC release from nanofibre mats were determined from the release curves against time *t*. The experimental drug release data were fitted to five typical mathematical models for drug release kinetics consisting of conventional zero-order model, first-order model, Higuchi model, Ritger–Peppas model, as well as Zeng model [20].

#### 2.4.1. Zero-Order Model

A zero-order model is employed to a drug delivery system in which the drug release rate is independent of drug concentration. Its empirical equation [25] can be described as

(2) $$\frac{M_{t}}{M_{\infty }}=K_{0}t$$

where *M_t_*, *M_∞_* and *K*_0_ are the absolute cumulative amounts by mass of drug released at time *t* and infinite time, and zero-order release constant, respectively. The ratio *M_t_*/*M_∞_* denotes the cumulative amount released in percentage [26].

#### 2.4.2. First-Order Model

The first-order equation can be expressed in a derived mathematical form [27] for the released drug in an aqueous phase [28] given below

(3) $$\frac{M_{t}}{M_{\infty }}=1-e^{K_{1}t}$$

in which *K*_1_ is the first-order release constant.

#### 2.4.3. Higuchi Model

Higuchi [29] developed important mathematical models to study the release of water-soluble and low soluble drugs loaded in semi-solid and solid matrices. The equation can be simplified by

(4) $$\frac{M_{t}}{M_{\infty }}=K_{H}t^{\frac{1}{2}}$$

where *K_H_* is Higuchi kinetic constant that reflects design variables in a drug delivery system. 

#### 2.4.4. Ritger–Peppas Model

Ritger and Peppas [25] established a simple exponential relationship to investigate both Fickian and non-Fickian drug release conditions in swelling and non-swelling polymeric delivery systems. Such a mathematical equation is given by

(5) $$\frac{M_{t}}{M_{\infty }}=K_{R}t^{n}$$

where *K_R_* is Ritger–Peppas kinetic constant, which incorporates structural and geometric characteristics of a macromolecular network system and the drug. *n* is the diffusion exponent for the transport mechanism through the polymer. 

#### 2.4.5. Zeng Model

Zeng and co-workers [20] developed a three-parameter model with the close-form analytical solution to consider the reversible drug–carrier interaction and first-order drug release from liposomes. The equation is relatively complex as given by

(6) $$\frac{M_{t}}{M_{\infty }}=\frac{K_{off}}{K_{on}+K_{off}}\left(1-e^{-K_{S}t}\right)+\frac{K_{on}}{K_{on}+K_{off}}\left(1-e^{-K_{off}t}\right)$$

and

(7) $$\Delta G=-k_{B}T\left(\frac{K_{on}}{K_{off}}\right)$$

In Equation (6), *K_on_* is the rate constant of association for non-dispersed drug molecules in the system to be disassociated from carriers prior to drug release. Conversely, *K_off_* is the rate constant of disassociation accordingly and *K_S_* is a constant proportional to the surface-to-volume ratio of the carriers in order to improve drug release. In addition, Δ*G* is the difference of free energy between the free and bound states, *k_B_* is the Boltzmann’s constant and *T* is the absolute temperature where *T* = 300 K in Equation (7). *K_off_*, *K_S_* and Δ*G* are three critical parameters in Zeng model to explain the effect of cumulative drug release.

### 2.5. In Vitro Biodegradation Study

With respect to in vitro degradation studies, nanofibre mats with the thickness ranging from 300 to 450 µm were cut into 2 cm × 2 cm testing samples. They were measured to determine the initial weight (*m*) and then were placed into an incubated rotary shaker at the rotor speed of 100 rpm and the temperature of 37 °C during the biodegradation study where 15 mL PBS (pH = 7.4) was utilised for the required incubation time.

Such nanofibre mats were removed at each designated incubation period and washed with deionised water. These mats were dried under vacuum at 37 °C until they reached a constant weight (*m*_1_). Mass loss (%) was determined using the equation below

(8) $$\mathrm{mass}\;\mathrm{loss}\;\left(\%\right)=\left(\frac{m-m_{1}}{m}\right)\times 100\%$$

## 3. Characterisation Techniques

The morphology of electrospun nanofibres was examined via an EVO 40XVP scanning electron microscope (SEM) (Carl Zeiss AG, Jena, Germany) at the accelerating voltage of 5 kV. Before SEM observation, the samples were sputter-coated with platinum. Fibre diameters were calculated from the SEM images by using an image analysis tool within Zeiss Smart SEM software. A minimum of 150 fibres was selected from multiple scanned SEM images for the measurements per sample based on a sampling rate of 15 fibres per image.

Solution viscosity was determined with the aid of a Visco 88 portable viscometer from Malvern Instruments, Malvern, UK. The electrical conductivity of the solution was measured by using a WP-81 Waterproof Conductivity Meter, TPS, Brendale, QLD, Australia).

XRD measurements of prepared samples were undertaken in a Bruker Discover 8 X-ray diffractometer, Bruker Corporation, Berlin, Germany. It operated at 40 kV and 40 mA using Cu-Kα radiation subjected to the monochromatisation with graphite sample monochromators in a 2*θ* range from 5° to 40° (scanning rate: 0.05°/s).

Thermal analysis was performed via a DSC6000 Perkin Elmer, Boston, MA, USA with a cryofill liquid nitrogen cooling system. Approximately 10 mg fibre mat was cut and sealed in an aluminium pan. The thermal behaviour was analysed during the first heating scan in a temperature range from −90 °C to 200 °C at the ramp rate of 10 °C/min.

Thermogravimetric analysis (TGA) was carried out by using a SeikoSII Exstar 6000 (TG/DTA 6200), Tokyo, Japan to evaluate the thermal decomposition effect of electrospun PLA:PCL fibres loaded with drugs. About 6–10 mg TGA samples were heated from 40 °C to 900 °C at a heating ramp rate of 10 °C/min under a nitrogen flow of 200 mL/min [30].

Fourier transform infrared spectroscopy (FTIR) was performed in a Spectrum 100 FTIR Spectrometer, Perkin Elmer, Kanagawa, Japan. Resulting spectra were recorded in a wavenumber range of 4000–550 cm^−1^ with the resolution of 4 cm^−1^ by using an attenuated total reflectance (ATR) technique [31].

The amount of TCH present in the release PBS was obtained by means of a UV-vis spectrophotometer, JascoV-67, Easton, MD, USA at two specific wavelengths of 360 nm [32] and 319 nm [33] for TCH and IMC drugs accordingly.

## 4. Results and Discussion

### 4.1. Drug Effect on Fiber Morphology

**Figure 2:**
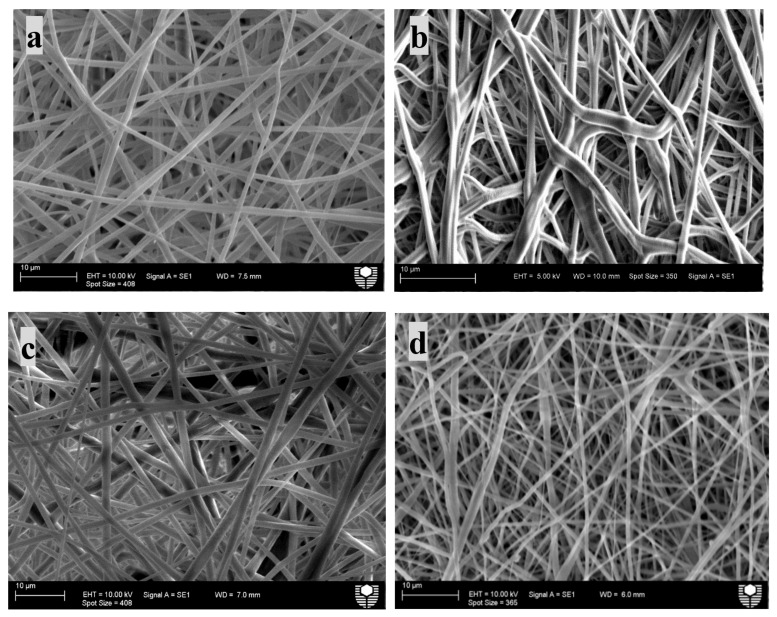

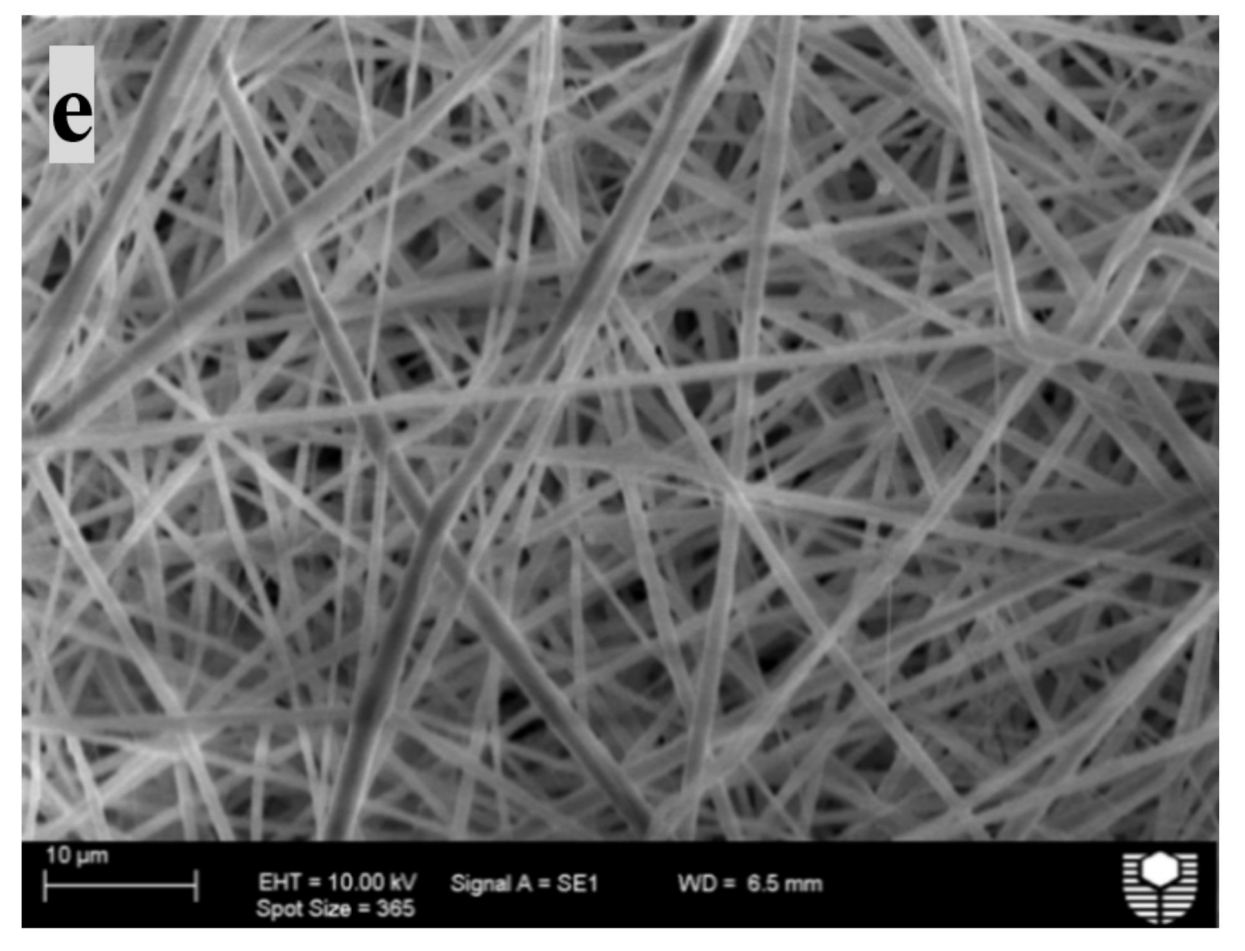
Scanning electron micrographs of electrospun fibre mats (**a**) PLA:PCL (**b**) PLA:PCL/HNT-ASP (**c**) PLA:PCL/IMC (**d**) PLA:PCL/TCH and (**e**) PLA:PCL/HNT-ASP /TCH. All the scale bars represent 10 µm in size.

**Figure 3:**
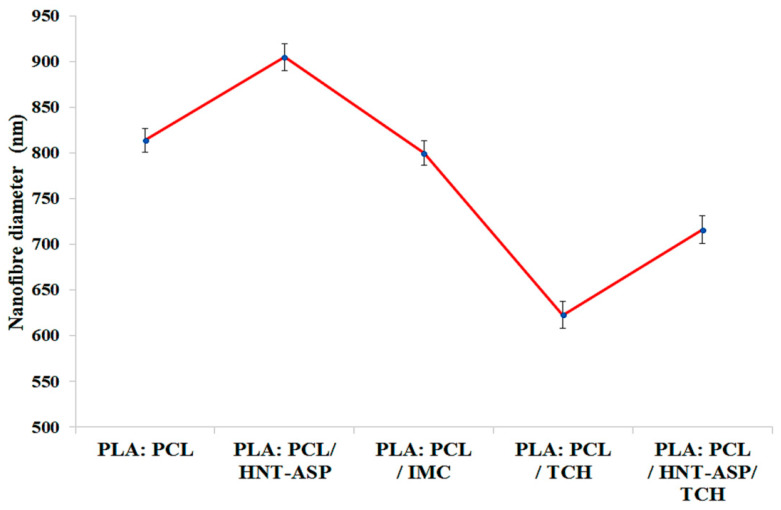
Effects of HNT-ASP, IMC and TCH on nanofibre diameter.

To investigate the drug effect, PLA:PCL polymer solution was blended with IMC and TCH at a fixed concentration of 5 wt%. As observed in Figure 2 and Figure 3, loaded IMC gave rise to uniform composite fibrous structures in possession of much larger fibre diameters at approximately 794 nm as opposed to 623 nm for TCH counterparts. No remarkable variation in fibre diameter was identified with the addition of IMC when compared with PLA:PCL blend fibres. Similarly, PLA:PCL/HNT-ASP/TCH composites were also found to attain uniform fibrous structures despite a small fibre diameter of 716 nm relative to those PLA:PCL blends and PLA:PCL/HNT-ASP composites. 

**Figure 4:**
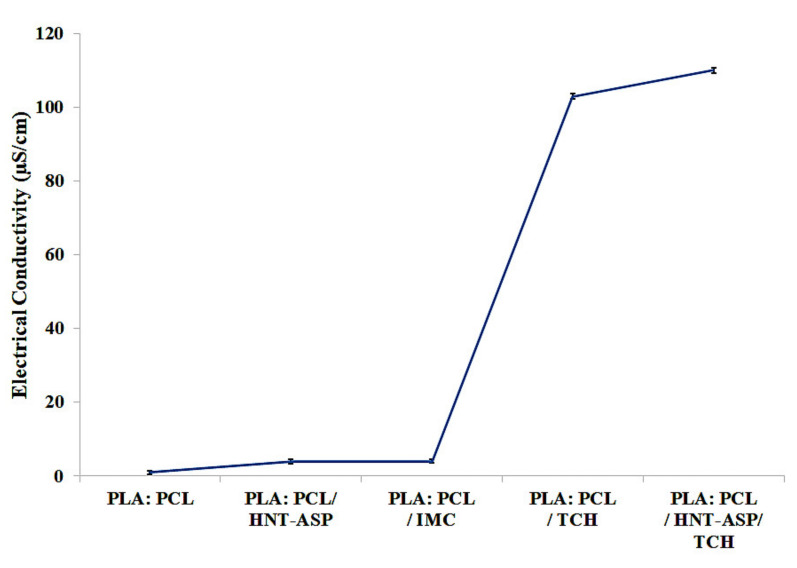
Effects of HNT-ASP, IMC and TCH on solution electrical conductivity.

Figure 4 reveals that the addition of IMC to PLA:PCL solution did not appear to significantly affect the solution electrical conductivity in good accordance with previous work [34]. However, as expected, the use of cationic drug TCH further increases the solution conductivity. The additional amphoteric molecules of TCH, having several ionisable functional groups [35], further increases the solution conductivity as compared to that without TCH.

### 4.2. Crystallinity Level

**Figure 5:**
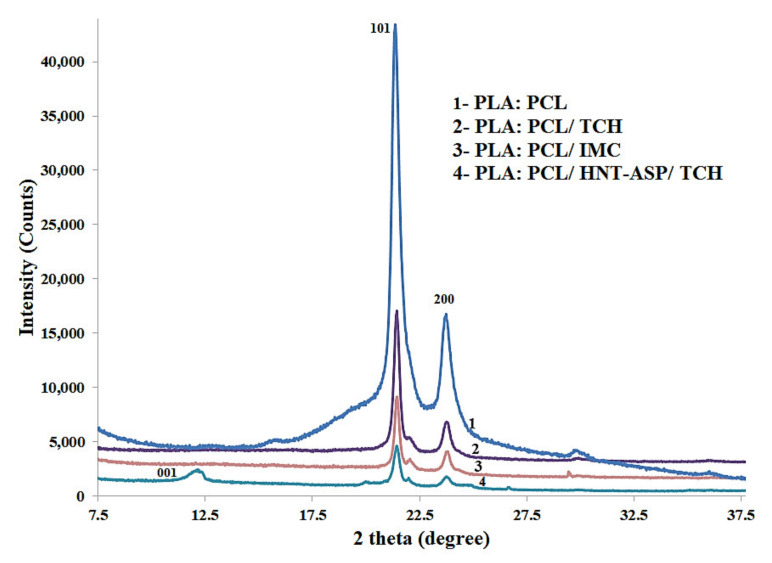
XRD patterns for selected material samples showing the relative positions of reflection peaks when loaded with TCH and IMC drugs.

An XRD examination was carried out to investigate the impact of drug type on crystallinity level and fibrous structures of electrospun nanocomposites fibre mats. The associated crystalline peaks in XRD patterns were directly detected with the aid of a DICVOL program used in the FullProf software [36] and labelled with XRD reflection and crystal planes (*hkl*) accordingly. Figure 5 illustrates the XRD patterns with respect to fibre mats of PLA:PCL, PLA:PCL/TCH, PLA:PCL/IMC and PLA:PCL/HNT-ASP/TCH. Such patterns possess two distinct diffraction peaks at the angles of 2*θ* = 20.1° and 23.2° corresponding to the crystal planes (101) and (200), respectively. In particular, the characteristic XRD peak of PCL/HNT-ASP/TCH was detected at 2*θ* = 12.4°, which is indicative of the HNT reflection plane (001). The basal reflection of HNTs results from their tubular morphological structures and small crystal size *L* = 21.7 nm obtained according to the Scherrer relation [8]. No significant difference in relation to peak position was manifested regardless of different material samples, indicating the minor effect arising from loaded TCH and IMC to alter crystalline structures of electrospun nanocomposite fibre mats. As such, it may be revealed that TCH and IMC are most likely to be dispersed in an amorphous state into PLA:PCL fibre mats. This may be attributed to rapid solvent evaporation, which did not have enough time to cause relaxation of chain orientation. A short period for drug recrystallisation and the formation of a preferred configuration in the amorphous state would take place accordingly [37].

**Figure 6:**
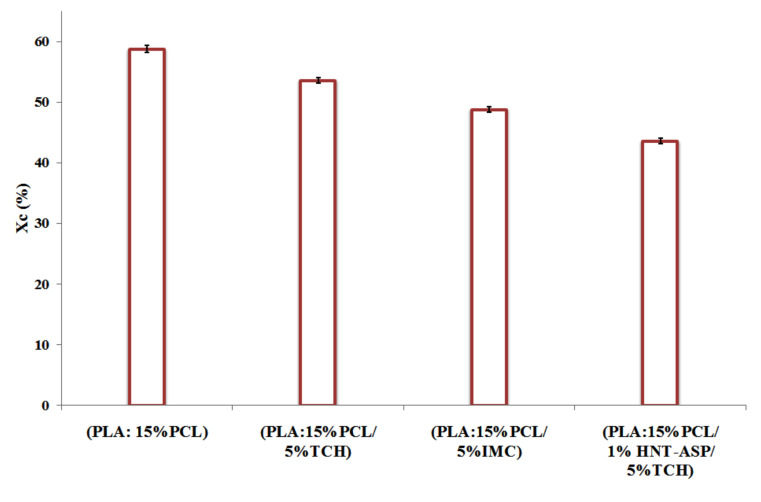
Degree of crystallinity (*X_c_*) of electrospun PLA: PCL fibre mats loaded with TCH and IMC drugs.

Figure 6 suggests that the degree of crystallinity (*X_c_*) of nanocomposite fibre mats is reduced more remarkably when TCH is concurrently added, as opposed to that of PLA: PCL counterparts. Additionally, the use of IMC loaded to PLA:PCL fibre mats was found to influence *X_c_* more significantly in comparison with TCH loaded counterparts. It is evidently shown that the presence of TCH and IMC enables to greatly accelerate the nucleation effect, thus resulting in a shorter period than the required time for the disentanglement of molecular chains. This is because the resulting degree of crystallinity can be impacted by the restricted mobility of polymeric chains in order to prevent the more rapid growth of developed crystals. In addition, the crystallinity of nanocomposites was reduced when HNT-ASP was embedded into polymers due to the diminished mobility of polymeric chains.

### 4.3. Thermal Properties

**Figure 7:**
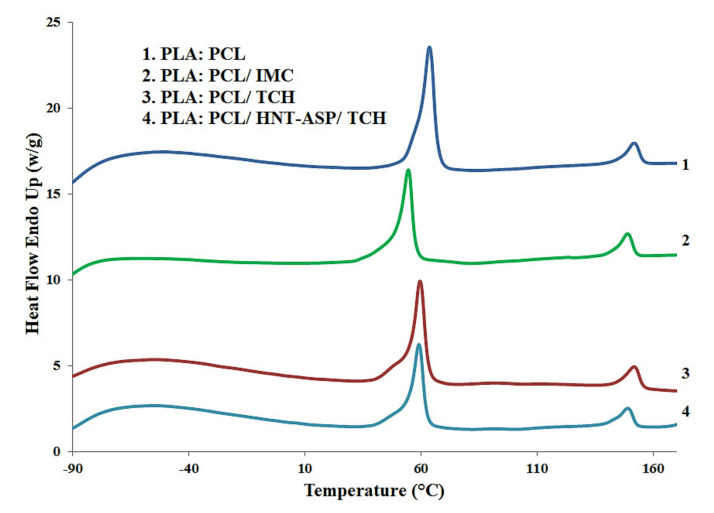
DSC thermograms for selected PLA:PCL material samples.

The thermal properties of electrospun nanocomposite fibre mats loaded with IMC and TCH separately via differential scanning calorimetry (DSC) are shown in Table 1 and Figure 7. There is a considerable decreasing trend with respect to the *T_g_* of PCL within nanocomposite fibre mats despite a slight decline for the *T_m_* levels of both PLA and PCL when embedded with HNT-ASP and drug-loaded with 5 wt%/v TCH. The effect of decreased *T_g_* can be associated with the low molecular weight of TCH. Short molecular chains of TCH are believed to yield the decline in the packing density of polymeric chains, thus facilitating the chain mobility as a result of lower *T_g_*. On the contrary, the addition of TCH offers a significant increase in *T_c_* with respect to electrospun nanocomposites. 

PLA:PCL/HNT-ASP/TCH fibre mats possess the crystallisation temperature (*T_c_*) about 103 °C as opposed to 106°C for PLA:PCL/TCH and 84 °C for PLA in PLA:PCL counterparts. This result suggests that low-molecular-weight TCH enables to hinder the cold crystallisation process of PLA in an anti-nucleating agent role as opposed to HNT-ASP. On the other hand, loaded IMC results in an evident drop in *T_m_* in contrast with a slight decline in *T_c_*. This finding lies in a better interaction between PLA:PCL fibre mats when compared with the use of TCH. The *T_g_* of PLA within the blends is hardly detected as it has overlapped the melting peak of PCL

### 4.4. TGA Analysis

**Figure 8:**
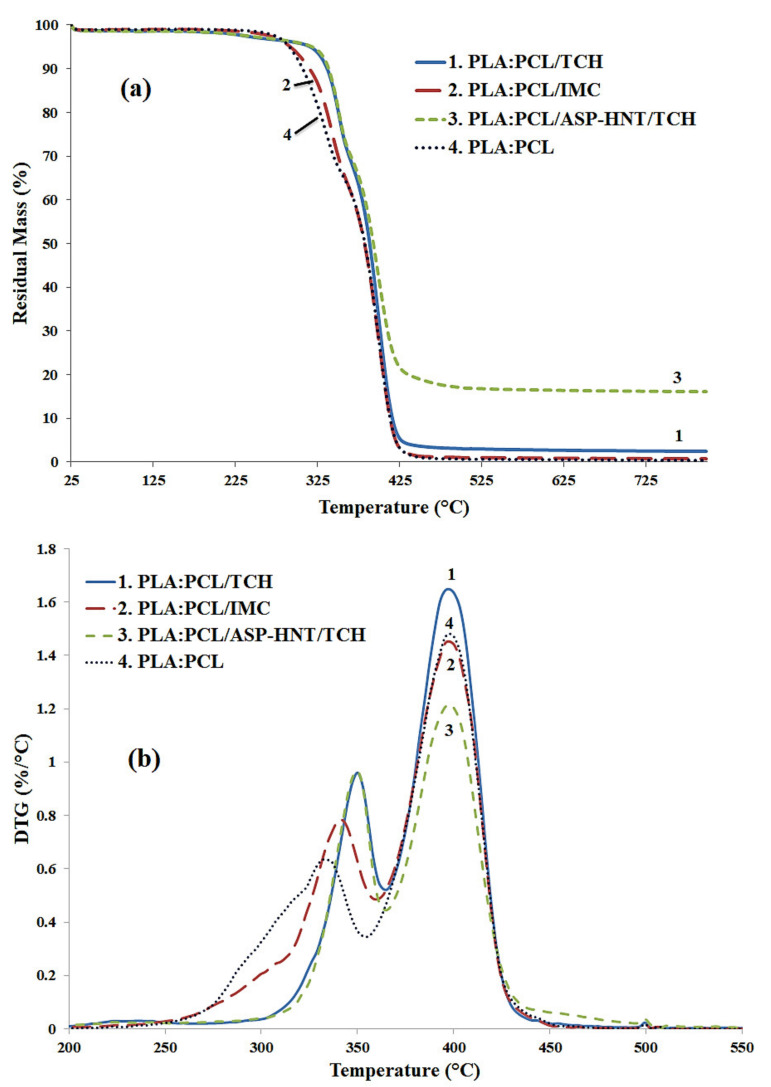
(**a**) TGA curves and (**b**) derivative thermogravimetric (DTGA) curves for typical PLA: PCL blend fibre mats.

TGA spectra of electrospun nanocomposite fibre mats loaded with IMC and TCH are exhibited in Figure 8. The addition of TCH, IMC or HNT-ASP does not appear to induce a significant alteration in thermal stability of PCL with a narrow degradation peak range from 398 to 399 °C. When TCH, IMC and HNT-ASP/TCH are incorporated, it is evidently demonstrated that a peak shift to higher temperatures takes place within nanocomposite fibre mats, thus retarding the thermal degradation. The temperature associated with the TGA peak, which is assigned to PLA at 334 °C within PLA:PCL fibre mats, shifts to 345 °C when loaded with IMC and further increases up to 350 °C with the addition of TCH and HNT-ASP/TCH, resulting in much better thermal stability. Furthermore, the residual masses were increasingly recorded to be 0.36%, 0.68%, 2.4% and 15.6% for PLA:PCL, PLA:PCL/IMC, PLA:PCL/TCH and PLA:PCL/HNT-ASP/TCH fibre mats, respectively. In particular, the higher residue mass of PLA:PCL/TCH when compared with that of PLA:PCL/IMC can be ascribed to different molecular structures of TCH (i.e., C_22_H_25_ClN_2_O_8_) with a larger number of carbon atoms relative to that of IMC (i.e., C_19_H_16_ClNO_4_). 

### 4.5. FTIR Evaluation

**Figure 9:**
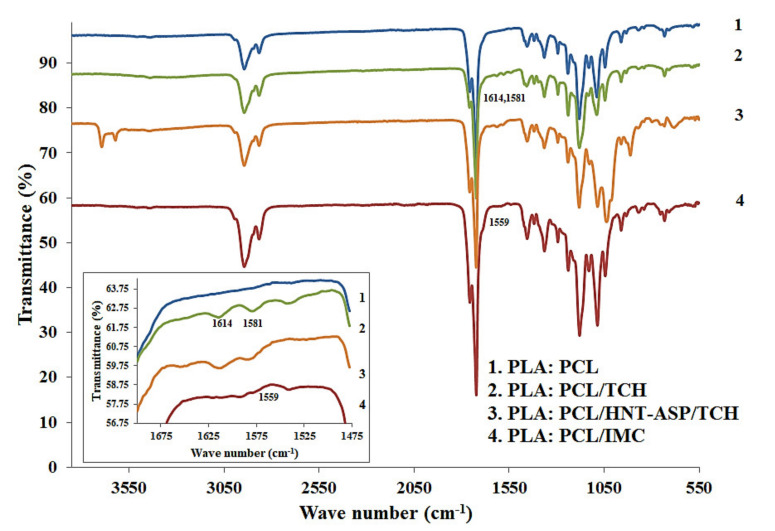
FTIR spectra for typical PLA:PCL based fibre mats showing drug effect on FTIR peaks.

Figure 9 depicts the FTIR spectra of PLA:PCL fibre mats loaded with or without TCH and IMC for comparison in terms of their chemical bonding effect. The spectra of TCH within PLA:PCL fibre mats and corresponding nanocomposite fibre mats do not appear to be easily assigned to the band shift. Nonetheless, corresponding TCH bands recorded at 1614 and 1581 cm^−1^ within nanocomposite fibre mats are assigned to C=O stretchings at ring A and ring C, respectively [38], signifying the successful encapsulation of TCH. In a similar manner, effective encapsulation of IMC is also manifested, as evidenced by the existing bond taking place at 1560 cm^−1^ associated with the ionisation of carboxyl groups [39] according to the FTIR spectra of PLA:PCL/IMC fibre mats. 

### 4.6. In Vitro Drug Release

**Figure 10:**
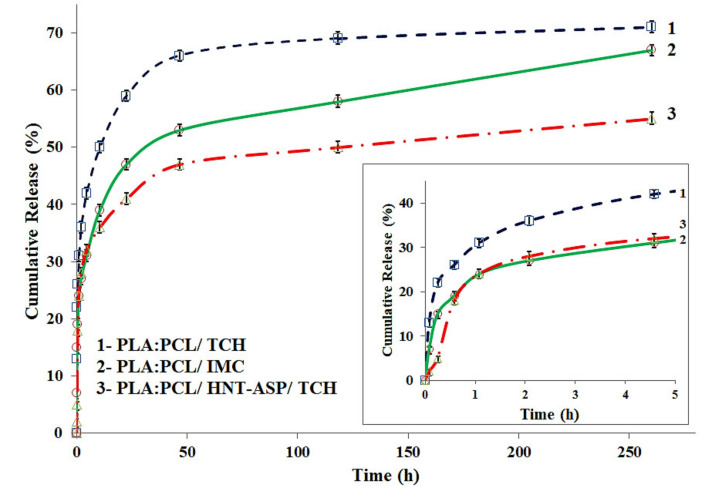
Drug release profiles for PLA:PCL fibre mats incorporated with IMC, TCH and HNT-ASP/TCH.

Drug particles within PLA:PCL fibre mats tend to remain on the fibre surfaces owing to the fast solvent evaporation arising from the corresponding blend solution in electrospinning along with high ionic interactions [40]. As such, it is quite convincing that a considerable burst release may happen at the initial drug-release stage. As a result of weak interaction, the burst release becomes more pronounced on the condition that it is incompatible between the drug and PLA:PCL solution. In comparison, the release rate of hydrophilic drug TCH, when interacting with hydrophobic PLA:PCL blends, becomes faster than that of hydrophobic drug IMC. As seen from Figure 10, drug release intends to be quite rapid during the first 5 h, which is especially the case when using TCH (i.e., 42% for PLA:PCL/TCH vs. 30% for PLA:PCL/IMC). This phenomenon is attributed to better interaction and more active compatibility taking place between IMC and PLA:PCL fibre mats, as opposed to the use of TCH. It is worth noting that chemical interaction between the drug and its carrier may impede drug crystallisation within its carrier leading to a sustained drug-release condition in a crystalline state [41]. The addition of 1 wt%/v HNT-ASP into PLA:PCL blends can reduce initial burst release to 30% after 5 h as opposed to initial 42% for PLA: PCL blends alone. In the meantime, a similar release trend was observed over the steady release period from 50 to 250 h, indicating a robust drug release control over both short and long evaluation periods, which appears to arise from the embedded HNT-ASP. It is well known that HNTs possess negative charges on the outer surfaces while positive charges on their inner surfaces [42]. Such distinct charges on HNT-ASP may give rise to the electrostatic interaction identified between TCH and outer surfaces, as well as the lumen structures of HNT-ASP. Accordingly, the drug release rate of TCH can be reduced when PLA:PCL/HNT-ASP nanocomposite fibre mats act as a drug carrier. 

### 4.7. Release Kinetics

The mechanism of drug release kinetics is vital to investigate the efficacy of drug release with the carriers. Consequently, our drug release data obtained were fitted with five mathematical models whose equations and corresponding parameters are explicitly listed in Table 2. The conventional first-order model reveals that drug release rate can completely depend on drug concentration and its carrier feature. Whereas, Ritger–Pappas model demonstrates a particular drug release mechanism following a Fickian transport phenomenon [26] according to *n* values in a dominant diffusion process. On the other hand, Zeng model [20] reveals that drug release may be significantly influenced by the diffusion process and close interaction between TCH and IMC drugs and PLA:PCL blend fibre mats as an effective carrier. In particular, IMC has relatively low free energy variation between free and bound states, namely Δ*G* shown in Table 2.It is found that Δ*G* = −2.1 × 10^−21^ for IMC when compared with Δ*G* = −2.0 × 10^−22^ for TCH. PLA:PCL fibre mats are supposed to decrease the release rate of hydrophobic IMC though it may not work for hydrophilic TCH owing to their enhanced interaction. Additionally, the decrease in *K_off_* values of IMC presented in Table 2 implies a strong drug-carrier interaction when compared with *K_off_* of TCH ranging from 0.0033 to 0.0025 h^−1^.

It is worth noting that with the incorporation of HNT-ASP into PLA:PCL fibre mats, very minor alteration is evidently observed in *K_S_* though Δ*G* could decrease from −2.0 × 10^−22^ to −2.2 × 10^−21^ J. This finding confirms that embedded HNT-ASP may decrease the drug release rate of TCH, and further overcome the fast release issue induced by the poor interaction between loaded TCH and PLA:PCL fibre mats. Generally speaking, the close interaction between TCH molecules and HNT-ASP enables to yield hydrogen bonds with silanol groups mounted on HNT-ASP. Since our proposed models do not take erosion/biodegradation and dimensional alteration of drug carriers into consideration, TCH release data are hard to be completely fitted with those available models. On the flip side, the fitting results based on Zeng model is seemingly in overall good agreement with those obtained through experimental data because this model detected the effect of interaction between both drugs (i.e., TCH and IMC) and the carrier (i.e., PLA: PCL nanofibres) on the release rate. 

### 4.8. Mass Loss of Fibre Mats 

**Figure 11:**
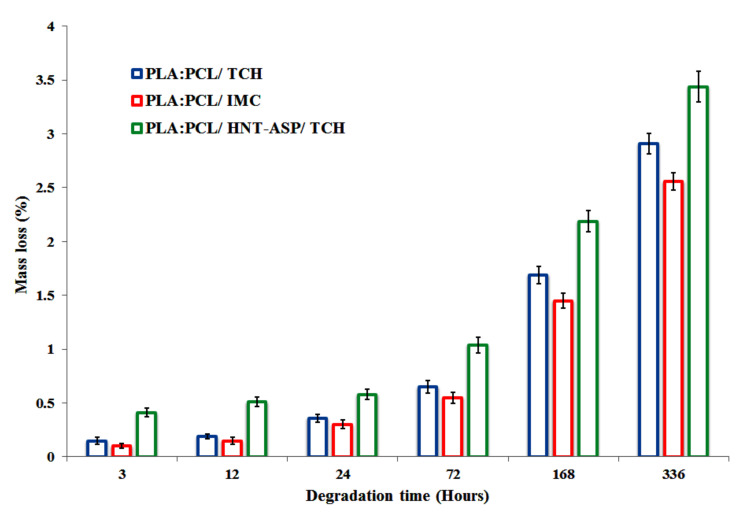
Mass loss of electrospun PLA:PCL fibre mats during both short and long degradation periods.

The biodegradability of PLA:PCL fibre mats in terms of mass loss is displayed in Figure 11. After the degradation period from the first 3 to 72 h in PBS, it is well noted that the weights of both PLA:PCL/TCH and PLA:PCL/IMC fibre mats have very trivial variations. The difference of mass has been identified between them to just commence after 168 h. It is worth mentioning that mass loss tends to increase over the degradation time up to 336 h by reaching the levels of 2.9% and 2.5% for PLA:PCL/TCH and PLA:PCL/IMC fibre mats, respectively. Overall, PLA:PCL/HNT-ASP/TCH nanocomposite fibre mats consistently gain the highest mass losses in all degradation time, as exemplified by 3.44% at 336 h. The small mass loss may be ascribed to PCL within the blends, thus hindering the water penetration inside the nanofibres. Additionally, water absorption has been found to take place slowly since PCL behaves with hydrophobic and semi-crystalline characteristics. 

In spite of smaller fibre diameters of PLA:PCL/TCH fibre mats, their degradation seems to be relatively high as compared with those of PLA:PCL/IMC counterparts. When hydrophilic drug TCH is released from fibre mats, it may accelerate the degradation process of hydrophobic nanofibres in a more rapid manner due to its increased wettability, as opposed to that of hydrophobic IMC drug. Moreover, HNTs have been detected to improve the PBS absorption and also increase the porosity at the nanofiber surfaces for PLA:PCL/HNT-ASP/TCH nanocomposite mats. Such a phenomenon presents the strong evidence for the good incorporation of TCH drug molecules onto HNT-ASP in order to reduce the drug release rate. However, such nanocomposite fibre mats may undergo relatively high degradability.

## 5. Conclusions

It was found that the addition of TCH appears to increase the electrical conductivity of PLA:PCL solution, thus considerably decreasing nanofiber diameters. However, nanofibre diameters are shown to be further altered insignificantly when loaded with IMC. Loaded TCH and IMC drugs help to decrease the degree of crystallinity, the *T_g_* and *T_m_* of PCL within PLA:PCL fibre mats. FTIR spectra confirm the successful encapsulation of IMC and TCH into PLA:PCL fibre mats and corresponding nanocomposites. Thermal degradation of PLA may be delayed with the addition of these two drugs, as well as HNT-ASP/TCH in nanocomposite fibre mats despite the little variation to PCL. When hydrophilic drug TCH is loaded into HNT-ASP and hydrophobic PLA:PCL blends, it decreases the drug release and overcomes the weak interaction between TCH and PLA:PCL blends. Such a typical characteristic is evidenced by excellent agreement achieved between Ritger–Peppas model and Zeng model and experimental data in order to clearly understand the mechanism of drug release kinetics. These models might not completely fit drug release data because they do not consider the erosion/biodegradation and dimensional alteration of the carriers. The mass loss in relation to the degradation effect signifies good TCH-embedding effect onto HNT-ASP leading to the reduction in drug release rate.

## Figures and Tables

**Table 1:** Thermal properties of PLA:PCL fibre mats and PLA:PCL nanocomposite fibre mats loaded with TCH and IMC.

Material Sample	*T_g_* (°C) PCL	*T_m_* (°C) PCL	*T_C_* (°C) PLA	*T_m_* (°C) PLA	*X_c_ (%)*
PLA:PCL	−52.19	63.75	83.97	152.54	58.84
PLA:PCL/IMC	−58.55	54.57	82.01	148.98	48.89
PLA:PCL/TCH	−58.48	59.64	105.77	151.93	53.69
PLA:PCL/HNT-ASP/TCH	−61.43	59.17	102.77	149.07	43.60

**Table 2:** Drug release parameters determined by fitting drug release data to five different mathematical models for drug release kinetics.

Mathematical Model	Zero-Order	First-Order	Higuchi	Ritger–Peppas	Zeng
**Equation**	***M_t_/M*_∞_ *= K_0_t***	***M_t_*/*M*_∞_ = 1 − *e*^−*K*^*_1_^t^***	***M_t_/M*_∞_ *= K_H_ t^1/2^***	***M_t_/M* *_∞_* *= K_R_t^n^***	**
PLA:PCL/IMC	*K*_0_ = 0.002 *R^2^* = 0.564	*K*_1_ = 0.436 *R^2^* = 0.822	*K_H_* = 0.037 *R^2^* = 0.801	*K_R_* = 1.618 *n* = 0.251 *R^2^* = 0.938	*K_on_ =* 0.0049 h^−1^ *K_off_* = 0.0025 h^−1^ *K_s_* = 1.006 h^−1^ * ΔG = -2.1 × 10^−21^J *R^2^* = 0.945
PLA:PCL/TCH	*K*_0_ = 0.002 *R^2^* = 0.434	*K*_1_ = 0.582 *R^2^* = 0.839	*K_H_* = 0.038 *R^2^* = 0.691	*K_R_* = 1.262 *n* = 0.204 *R^2^* = 0.927	*K_on_* = 0.0034 h^−1^ *K_off_* = 0.0033 h^–1^ *K_s_* = 1.160 h^−1^ * ΔG = −2.0 × 10^−22^J *R^2^* = 0.920
PLA:PCL/ HNT-ASP / TCH	*K*_0_ = 0.0012 *R^2^* = 0.314	*K*_1_ = 0.857 *R^2^* = 0.961	*K_H_* = 0.024 *R^2^* =0.534	*K_R_* = 2.025 *n* = 0.323 *R^2^* = 0.684	*K_on_* = 0.0012 h^−1^ *K_off_* = 0.00068 h^−1^ *K_s_* = 1.025 h^−1^ * ΔG = -2.2×10^−21^J *R^2^* = 0.986

** *M_t_*/*M_∞_* = (*K_off_*/(*K_on_* + *K_off_*))(1 − *e^−K^_S_^t^*) + (*K_on_*/(*K_on_* + *K_off_*))(1 − *e^−K^_off_^t^*). * Δ*G* *= −k_B_T* ln(*K_on_/K_off_*) where *k_B_* is the Boltzmann’s constant and *T* is the absolute temperature (300 K).

## Data Availability

Data is contained within the article.

## References

[1] Agarwal S., Wendorff J., Greiner A. (2008). Use of electrospinning technique for biomedical applications. Polymer.

[2] He S.-W., Li S.-S., Hu Z.-M., Yu J.-R., Chen L., Zhu J. (2011). Effects of three parameters on the diameter of electrospun poly (ethylene oxide) nanofibers. J. Nanosci. Nanotechnol..

[3] Han J., Chen T., Branford-White C., Zhu L. (2009). Electrospun shikonin-loaded PCL/PTMC composite fiber mats with potential biomedical applications. Int. J. Pharm..

[4] Haroosh H.J., Dong Y., Lau K.-T. (2014). Tetracycline hydrochloride (TCH)-loaded drug carrier based on PLA: PCL nanofibre mats: Experimental characterisation and release kinetics modelling. J. Mater. Sci..

[5] Bi H., Feng T., Li B., Han Y. (2020). In Vitro and In Vivo Comparison Study of Electrospun PLA and PLA/PVA/SA Fiber Membranes for Wound Healing. Polymers.

[6] Du M., Guo B., Lei Y., Liu M., Jia D. (2008). Carboxylated butadiene-styrene rubber/halloysite nanotube nanocomposites: Interfacial interaction and performance. Polymer.

[7] Haroosh H.J., Dong Y., Ingram G.D. (2013). Synthesis, Morphological Structures, and Material Characterization of Electrospun PLA: PCL/Magnetic Nanoparticle Composites for Drug Delivery. J. Polym. Sci. Part B Polym. Phys..

[8] Haroosh H.J., Dong Y., Chaudhary D.S., Ingram G.D., Yusa S. (2013). Electrospun PLA: PCL composites embedded with unmodified and 3-aminopropyltriethoxysilane (ASP) modified halloysite nanotubes (HNT). Appl. Phys. A Mater. Sci. Process..

[9] Kenawy E., Abdel-Hay F., El-Newehy M., Wnek G. (2009). Processing of polymer nanofibers through electrospinning as drug delivery systems. Mater. Chem. Phys..

[10] Kweon H.Y., Yoo M.K., Park I.K., Kim T.H., Lee H.C., Lee H.S., Oh J.S., Akaike T., Cho C.S. (2003). A novel degradable polycaprolactone networks for tissue engineering. Biomaterials.

[11] Sun M., Downes S. (2009). Physicochemical characterisation of novel ultra-thin biodegradable scaffolds for peripheral nerve repair. J. Mater. Sci. Mater. Med..

[12] Deng X.L., Sui G., Zhao M.L., Chen G.Q., Yang X.P. (2007). Poly (L-lactic acid)/hydroxyapatite hybrid nanofibrous scaffolds prepared by electrospinning. J. Biomater. Sci. Polym. Ed..

[13] Haroosh H.J., Chaudhary D.S., Dong Y. (2012). Electrospun PLA/PCL fibers with tubular nanoclay: Morphological and structural analysis. J. Appl. Polym. Sci..

[14] Leonés A., Peponi L., Lieblich M., Benavente R., Fiori S. (2020). In Vitro Degradation of Plasticized PLA Electrospun Fiber Mats: Morphological, Thermal and Crystalline Evolution. Polymers.

[15] Lv G., He F., Wang X., Gao F., Zhang G., Wang T., Jiang H., Wu C., Guo D., Li X. (2008). Novel nanocomposite of nano Fe3O4 and polylactide nanofibers for application in drug uptake and induction of cell death of leukemia cancer cells. Langmuir.

[16] Kim G., Yoon H., Park Y. (2010). Drug release from various thicknesses of layered mats consisting of electrospun polycaprolactone and polyethylene oxide micro/nanofibers. Appl. Phys. A Mater. Sci. Process..

[17] Rezk A.I., Kim K.-S., Kim C.S. (2020). Poly (ε-Caprolactone)/Poly (Glycerol Sebacate) Composite Nanofibers Incorporating Hydroxyapatite Nanoparticles and Simvastatin for Bone Tissue Regeneration and Drug Delivery Applications. Polymers.

[18] Kupka V., Dvořáková E., Manakhov A., Michlíček M., Petruš J., Vojtová L., Zajíčková L. (2020). Well-blended PCL/PEO electrospun nanofibers with functional properties enhanced by plasma processing. Polymers.

[19] Xie Z., Buschle Diller G. (2010). Electrospun poly (D, L lactide) fibers for drug delivery: The influence of cosolvent and the mechanism of drug release. J. Appl. Polym. Sci..

[20] Zeng L., An L., Wu X. (2011). Modeling Drug-Carrier Interaction in the Drug Release from Nanocarriers. J. Drug Deliv..

[21] Barani H., Khorashadizadeh M., Haseloer A., Klein A. (2020). Characterization and release behavior of a thiosemicarbazone from electrospun polyvinyl alcohol core-shell nanofibers. Polymers.

[22] Xu X., Zhong W., Zhou S., Trajtman A., Alfa M. (2010). Electrospun PEG–PLA nanofibrous membrane for sustained release of hydrophilic antibiotics. J. Appl. Polym. Sci..

[23] Buschle-Diller G., Cooper J., Xie Z., Wu Y., Waldrup J., Ren X. (2007). Release of antibiotics from electrospun bicomponent fibers. Cellulose.

[24] Chennamaneni S., Zhong B., Lama R., Su B. (2012). COX inhibitors Indomethacin and Sulindac derivatives as antiproliferative agents: Synthesis, biological evaluation, and mechanism investigation. Eur. J. Med. Chem..

[25] Ritger P.L., Peppas N.A. (1987). A simple equation for description of solute release I. Fickian and non-fickian release from non-swellable devices in the form of slabs, spheres, cylinders or discs. J. Control. Release.

[26] Cai X., Luan Y., Dong Q., Shao W., Li Z., Zhao Z. (2011). Sustained release of 5-fluorouracil by incorporation into sodium carboxymethylcellulose sub-micron fibers. Int. J. Pharm..

[27] Das R.K., Kasoju N., Bora U. (2010). Encapsulation of curcumin in alginate-chitosan-pluronic composite nanoparticles for delivery to cancer cells. Nanomed. Nanotechnol. Biol. Med..

[28] Chittur K.K. (1998). FTIR/ATR for protein adsorption to biomaterial surfaces. Biomaterials.

[29] Higuchi T. (1963). Mechanism of sustained-action medication. Theoretical analysis of rate of release of solid drugs dispersed in solid matrices. J. Pharm. Sci..

[30] Dong Y., Ghataura A., Takagi H., Haroosh H.J., Nakagaito A.N., Lau K.-T. (2014). Polylactic acid (PLA) biocomposites reinforced with coir fibres: Evaluation of mechanical performance and multifunctional properties. Compos. Part A Appl. Sci. Manuf..

[31] Andrade J., Pereira C.G., de Almeida Junior J.C., Viana C.C.R., de Oliveira Neves L.N., da Silva P.H.F., Bell M.J.V., dos Anjos V.d.C. (2019). FTIR-ATR determination of protein content to evaluate whey protein concentrate adulteration. Lwt.

[32] Kenawy E., Bowlin G., Mansfield K., Layman J., Simpson D., Sanders E., Wnek G. (2002). Release of tetracycline hydrochloride from electrospun poly (ethylene-co-vinylacetate), poly (lactic acid), and a blend. J. Control. Release.

[33] Mi F.L., Sung H.W., Shyu S.S. (2001). Release of indomethacin from a novel chitosan microsphere prepared by a naturally occurring crosslinker: Examination of crosslinking and polycation–anionic drug interaction. J. Appl. Polym. Sci..

[34] Nyström M., Murtomaa M., Salonen J. (2010). Fabrication and characterization of drug particles produced by electrospraying into reduced pressure. J. Electrost..

[35] Sassman S.A., Lee L.S. (2005). Sorption of three tetracyclines by several soils: Assessing the role of pH and cation exchange. Environ. Sci. Technol..

[36] Boultif A., Louer D. (2004). Powder pattern indexing with the dichotomy method. J. Appl. Crystallogr..

[37] Puppi D., Piras A., Detta N., Dinucci D., Chiellini F. (2010). Poly (lactic-co-glycolic acid) electrospun fibrous meshes for the controlled release of retinoic acid. Acta Biomater..

[38] Li Z., Kolb V.M., Jiang W.-T., Hong H. (2010). Ftir and XRD INVestigations of tetracycline intercalation in smectites. Clays Clay Miner..

[39] Mohanambe L., Vasudevan S. (2005). Anionic clays containing anti-inflammatory drug molecules: Comparison of molecular dynamics simulation and measurements. J. Phys. Chem. B.

[40] He C., Huang Z., Han X., Liu L., Zhang H., Chen L. (2006). Coaxial electrospun poly (l-lactic acid) ultrafine fibers for sustained drug delivery. J. Macromol. Sci. Part B.

[41] Natu M., de Sousa H., Gil M. (2010). Effects of drug solubility, state and loading on controlled release in bicomponent electrospun fibers. Int. J. Pharm..

[42] Vergaro V., Abdullayev E., Lvov Y.M., Zeitoun A., Cingolani R., Rinaldi R., Leporatti S. (2010). Cytocompatibility and uptake of halloysite clay nanotubes. Biomacromolecules.

